# Unraveling the molecular heterogeneity in type 2 diabetes: a potential subtype discovery followed by metabolic modeling

**DOI:** 10.1186/s12920-020-00767-0

**Published:** 2020-08-24

**Authors:** Maryam Khoshnejat, Kaveh Kavousi, Ali Mohammad Banaei-Moghaddam, Ali Akbar Moosavi-Movahedi

**Affiliations:** 1 Laboratory of Complex Biological Systems and Bioinformatics (CBB), Department of Bioinformatics, Institute of Biochemistry and Biophysics (IBB), University of Tehran, Tehran, Iran; 2 The UNESCO Chair on Interdisciplinary Research in Diabetes, Institute of Biochemistry and Biophysics (IBB), University of Tehran, Tehran, Iran; 3 Laboratory of Genomics and Epigenomics (LGE), Department of Biochemistry, Institute of Biochemistry and Biophysics (IBB), University of Tehran, Tehran, Iran; 4 Institute of Biochemistry and Biophysics, University of Tehran, Tehran, Iran

**Keywords:** Type 2 diabetes, Subtype, Classification, Clustering, Flux variability analysis, Muscle, Insulin resistance, Metabolic modeling

## Abstract

**Background:**

Type 2 diabetes mellitus (T2DM) is a complex multifactorial disease with a high prevalence worldwide. Insulin resistance and impaired insulin secretion are the two major abnormalities in the pathogenesis of T2DM. Skeletal muscle is responsible for over 75% of the glucose uptake and plays a critical role in T2DM. Here, we sought to provide a better understanding of the abnormalities in this tissue.

**Methods:**

The muscle gene expression patterns were explored in healthy and newly diagnosed T2DM individuals using supervised and unsupervised classification approaches. Moreover, the potential of subtyping T2DM patients was evaluated based on the gene expression patterns.

**Results:**

A machine-learning technique was applied to identify a set of genes whose expression patterns could discriminate diabetic subjects from healthy ones. A gene set comprising of 26 genes was found that was able to distinguish healthy from diabetic individuals with 94% accuracy. In addition, three distinct clusters of diabetic patients with different dysregulated genes and metabolic pathways were identified.

**Conclusions:**

This study indicates that T2DM is triggered by different cellular/molecular mechanisms, and it can be categorized into different subtypes. Subtyping of T2DM patients in combination with their real clinical profiles will provide a better understanding of the abnormalities in each group and more effective therapeutic approaches in the future.

## Background

T2DM is a complex multifactorial disorder. Impaired insulin secretion by pancreatic β-cells is the main cause of T2DM. This usually happens due to having a background of reduced sensitivity to insulin in target tissues [1]. Skeletal muscle, liver, and adipose tissues are the key insulin-sensitive tissues. Skeletal muscle takes a major role in lowering the blood glucose level and is responsible for over 75% of the glucose uptake [2, 3]. Better prognostic signatures and therapeutic targets necessitate a better understanding of the molecular mechanisms underlying insulin resistance in skeletal muscle. Whereas considerable experimental and computational attempts have been made to determine the molecular mechanisms involved in insulin resistance [4–8], the exact underlying cause of this phenomenon is still unclear [4], and in some cases, failure of the current therapies has been reported. One possible reason for this failure may be the multifactorial nature of T2DM, which results in different groups of molecular mechanisms, all leading to insulin resistance. Precision medicine for each group may, therefore, help develop more effective treatments for T2DM.

**Fig. 1:**
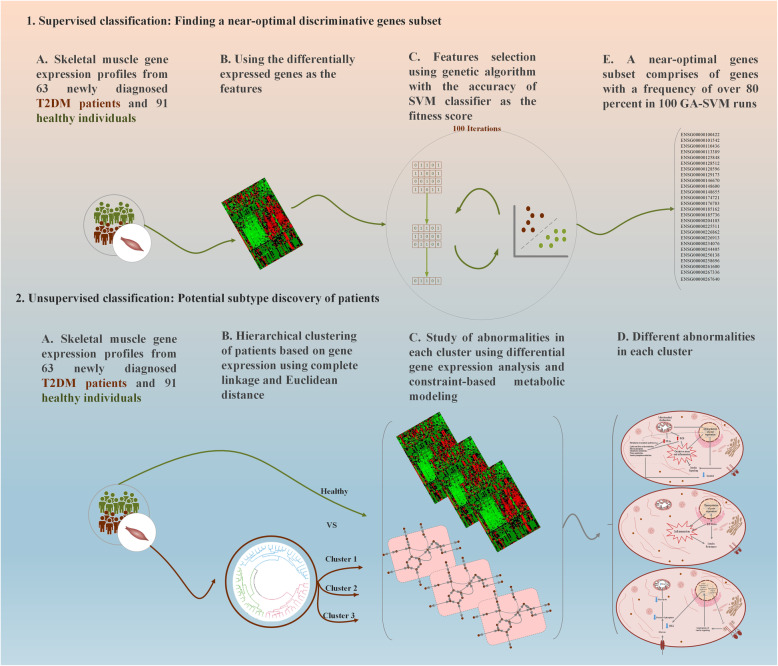
Graphical overview of the study design. This study included two supervised and unsupervised classification sections. At the supervised classification part, we used a machine learning approach to identify a set of genes whose expression patterns could discriminate T2DM individuals from healthy ones. At the unsupervised section, the clustering of T2DM patients was employed for potential subtyping of the disease

In the present work, we attempted to better understand T2DM. We studied gene expression profiles of human skeletal muscle from healthy and newly diagnosed diabetic patients with two goals: 1) To identify a set of genes whose expression patterns can discriminate T2DM individuals from healthy ones using a machine-learning approach; and 2) To examine the potential existence of molecular subtypes based on the gene expression profile of diabetic individuals. For this purpose, unsupervised classification was used to find different possible subgroups of T2DM. We applied differential gene expression analysis and metabolic modeling to gain an in-depth insight into the molecular mechanisms leading to insulin resistance in each subgroup. This finding can be helpful in developing effective treatments for this disease in the future. The overall study design is shown in Fig. 1.

## Methods

### Data

Gene expression data were obtained from a sub-study of the Finland-United States Investigation of NIDDM Genetics project [9]. This is the largest dataset of human skeletal muscle transcriptome. The dataset contains gene expression data from participants with glucose tolerance ranging from normal to newly diagnosed T2DM, in which 91 and 63 individuals were healthy and diabetic, respectively. Data are available through the repository’s data access request procedure in the database of Genotypes and Phenotypes (dbGaP) with the accession code phs001068.v1.p1. Data from healthy and diabetic individuals were downloaded and were used for subsequent analyses.

### Differential gene expression

Detection of differentially expressed genes (DEGs) was done by employing the DESeq2, which is a standard, well-known, and powerful method for RNA-Seq differential gene expression analysis, and gives the highest power estimations even with a small sample size [10, 11]. The analysis was conducted using the Bioconductor R package DESeq2 [12]. A pre-filtering stage was performed that removed genes whose expression levels were below a minimum cutoff level (< 5 read counts in less than 25% of samples). According to the DESeq2 manual, between samples normalization was applied to account for differences in sequencing depth. DESeq2 employs one of the best between-sample normalization methods to detect differentially expressed genes [13]. Since without between sample normalization in RNA-Seq data, cross-sample analysis is not reliable, we did not use FPKM/RPKM in this analysis. These are only suitable for the comparison of genes in one sample. In the context of differential analysis, RPKM [FPKM] is inefficient and should be abandoned [13].

DEGs between two states (e.g., healthy vs. diabetic) were assessed based on a negative binomial distribution. Multiple testing correction was applied by adjusting the *P* values using the Benjamini–Hochberg procedure and false discovery rate of 0.1 was considered significant. Moreover, the KEGG pathway enrichment analysis of significant DEGs was performed using Enrichr [14]. For this enrichment analysis, we used the genes with the absolute value of log2 fold change more than 0.9.

### Feature selection method: GA–SVM

To select a near-optimal feature subset, a wrapper feature selection algorithm that is a hybrid of genetic algorithm (GA) and support vector machine (SVM), was used. GA is a global optimal search algorithm inspired by Darwin’s theory of evolution. In the algorithm, the candidate solution (feature subset) is encoded on a chromosome-like structure. A set of chromosomes constitutes a population in which crossover and mutation can occur to generate new feature subsets. For each chromosome, a fitness value is calculated representing how well a feature subset is adapted to the environment. The algorithm employs a competing solution in which better feature subsets have more chance to be selected for reproduction and creating the next generation. This search process will be repeated until a stopping criterion is satisfied.

In this analysis, a binary genetic algorithm was implemented. Each gene in this algorithm has one of the binary values, 1 or 0, as either the presence or absence of a particular feature at the relevant chromosome. The chromosome length and the population size were set to the number of features and 500 chromosomes, respectively. The maximal number of generations was set to be 100. SVM classification accuracy was used as the fitness score. The genetic algorithm was terminated when the fitness score was at least 95% or the maximum number of generations was reached.

### Supervised classification

Supervised machine learning methods, including SVM, k-nearest neighbor (KNN), neural network (NN), naïve Bayes (NB), and random forest (RF) were employed for the classification of T2DM individuals from controls. We used the Orange data mining toolbox for this analysis [15]. The classifiers were validated by 10-fold stratified cross-validation and analysis of the area under the ROC curve (AUC), accuracy (ACC), F1 score, precision, and recall were reported.

### Unsupervised classification

Potential subtyping of diabetic patients was performed. The gene expression values were considered as the features for unsupervised classification. The low expressed genes were filtered and the remaining genes were normalized using the DESeq2 normalization method. This resulted in 21,826 genes as the features. Samples were categorized into potential subtypes based on the similarity in their gene expression patterns. Here, we used complete linkage hierarchical clustering with the Euclidean distance metric.

### Cluster-based genome-scale metabolic modeling

To reconstruct the personalized metabolic model, we need a generic genome-scale metabolic model (GEM) and gene expression data. A generic human GEM is reconstructed from all possible reactions, in which relevant enzymes are encoded in the genome, and can occur in different human cell types. By having gene-protein-reaction associations and mapping gene expression data to the generic metabolic model, active enzymes and subsequently active reactions are identified, and a context-specific metabolic model will be reconstructed. These context-specific metabolic models can be employed for subsequent simulations to study metabolic reprogramming under specific conditions. Possible minimum and maximum flux through a specific reaction can be simulated using flux variability analysis (FVA). The readers are referred to [16] for a full description of the principle concept of this simulation.

**Fig. 2:**
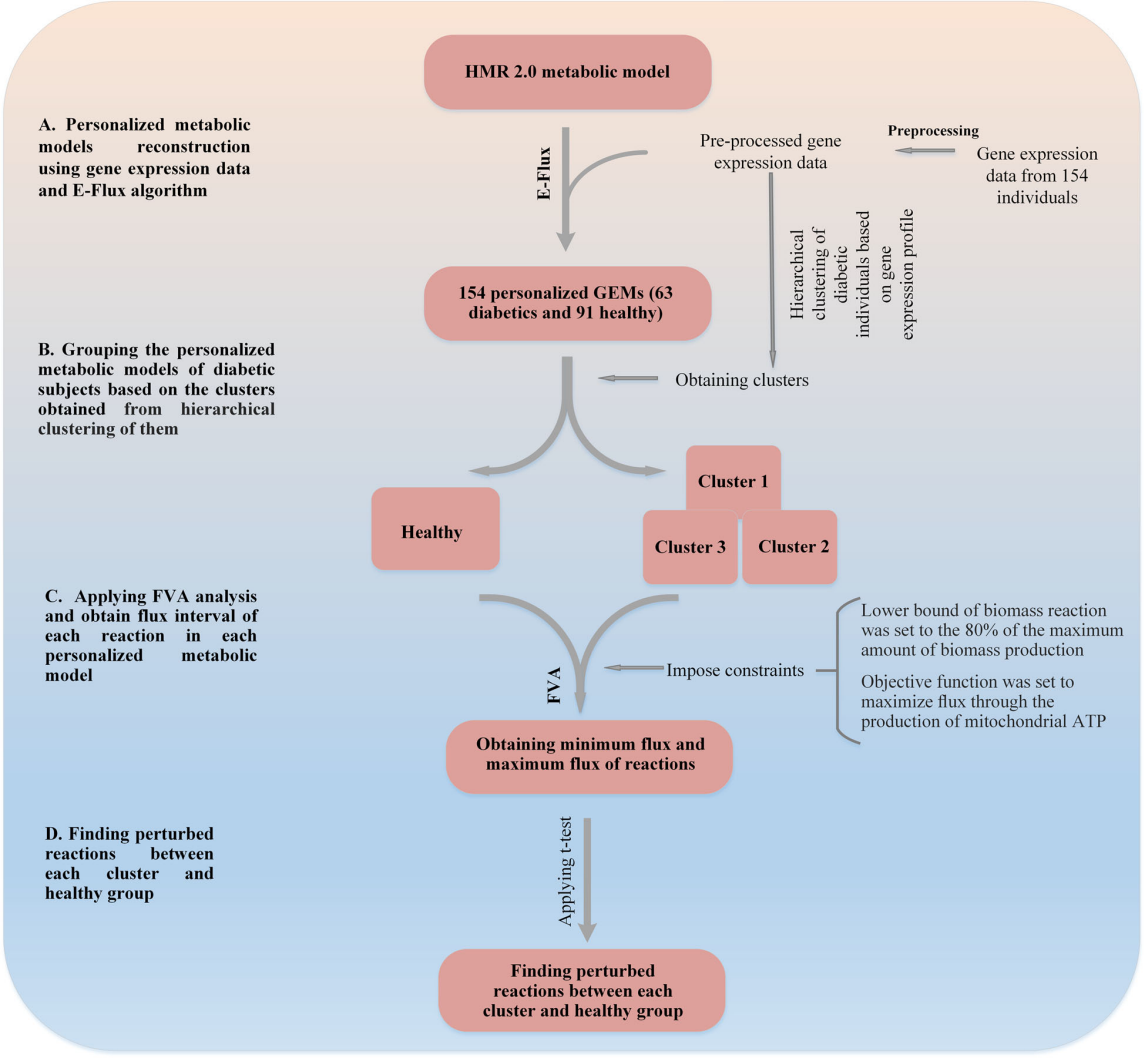
Workflow for cluster-based metabolic modeling. HMR2 model was used as the generic model. The personalized metabolic models were reconstructed by integrating gene expression data into the HMR2 using the E-Flux algorithm. Diabetic models were categorized into three groups based on the clusters obtained from the hierarchical clustering of T2DM patients. FVA was employed to obtain maximum and minimum possible fluxes in each reaction. Perturbed reactions in each cluster in comparison to the healthy group were identified by applying t-test on obtained fluxes

Here, personalized metabolic models were reconstructed based on the Human Metabolic Reaction 2 (HMR 2) as the generic model [17, 18]. E-Flux method was applied to reconstruct the context-specific metabolic models, using gene expression data [19]. Pre-processing of gene expression data, including pre-filtering of low expressed genes, between-sample normalization (DESeq2 normalization method with gene length adjustment), and log2 transformation was applied. The myocyte biomass reaction was added to the model from the Bordbar model [6]. Body fluid metabolites were used as media conditions [20]. The objective function was set to maximize flux through the production of mitochondrial ATP. Besides, to ensure the viability of the cell, the lower bound of biomass reaction was set to 0.8 of the maximum amount of biomass production in the healthy model [21]. FVA for each model was applied to obtain the minimum and maximum possible fluxes of each reaction using the COBRA Toolbox version 3.0 [22]. Personalized metabolic models (154 models) were categorized into the three groups based on the clusters obtained from the previous section. Subsequently, to find perturbed reactions between each cluster and controls, a two-sample t-test was performed on the minimum and maximum fluxes obtained from FVA. Multiple testing correction was applied using the Benjamini–Hochberg procedure, and reactions with false discovery rate less than 0.1 were considered as significant perturbed reactions. Figure 2 shows the workflow for this section.

## Results

### Supervised classification

There were 57,820 gene expression values for each individual that can be regarded as features in the classification. Using all of these genes as features were not applicable, leading to the high dimensional data and reduced performance of the conventional machine learning approaches. To overcome this problem, we used differential gene expression analysis. We removed those genes without any significant quantitative changes in T2DM versus healthy group from the feature list. Regarding the remaining genes as features, we applied a feature selection method to find near-optimal genes subset whose expression patterns can discriminate T2DM individuals from healthy ones. Thus, DEGs between healthy and T2DM were explored, which resulted in 247 differentially expressed genes. These 247 genes were used as the features of classification, and classifiers’ accuracy was investigated. SVM, KNN, NN, NB, and RF classifiers were evaluated and SVM showed the best performance in our analysis, as are shown in Table 1.

**Table 1:** Evaluation of the different classifiers for discrimination of T2DM individuals from healthy ones. Here, 247 differentially expressed genes were used as the classification features

Method	AUC	ACC	F1	Precision	Recall
**SVM**	0.889	0.838	0.806	0.788	0.825
**NN**	0.877	0.812	0.772	0.766	0.778
**RF**	0.837	0.766	0.71	0.721	0.698
**NB**	0.801	0.734	0.717	0.634	0.825
**KNN**	0.758	0.727	0.58	0.784	0.46

AUC, ACC, F1 score, precision, and Recall are reported

To achieve a near-optimal feature subset and to improve the classification accuracy, feature selection was applied based on a combination of GA and SVM. Different subsets of features were found that could distinguish T2DM from normoglycemic subjects with high accuracy. The GA-SVM procedure was repeated 100 times and 100 feature subsets with the prediction accuracy around 95 percentages were obtained. Features were ranked according to the frequency of their presence in these 100 subsets. Our analysis revealed that using 26 top-ranked genes as the features could improve classification accuracy to 94%. This subset consists of important genes including, *CERK*, *FGFBP3*, *ETV5*, *E2F8*, *MAFB*, and ten non-coding genes. The complete list of genes with Ensemble ID can be found in Additional file 2. These top-ranked genes were selected as the final features. The performance of different classifiers with these features was assessed (Table 2).

**Table 2:** Performance of different classifiers when the 26 top-ranked genes were used as the features

Method	AUC	ACC	F1	Precision	Recall
**SVM**	0.958	0.942	0.927	0.950	0.905
**NN**	0.966	0.903	0.878	0.900	0.857
**NB**	0.896	0.818	0.791	0.746	0.841
**RF**	0.836	0.799	0.735	0.796	0.683
**KNN**	0.829	0.721	0.538	0.833	0.397

To evaluate the SVM classifier using final features, classification was repeated 100 times with 10-fold cross-validation, and accuracy, sensitivity, and specificity were calculated. Figure S1 in Additional file 1 shows the box plot of this evaluation.

### Unsupervised classification

**Fig. 3:**
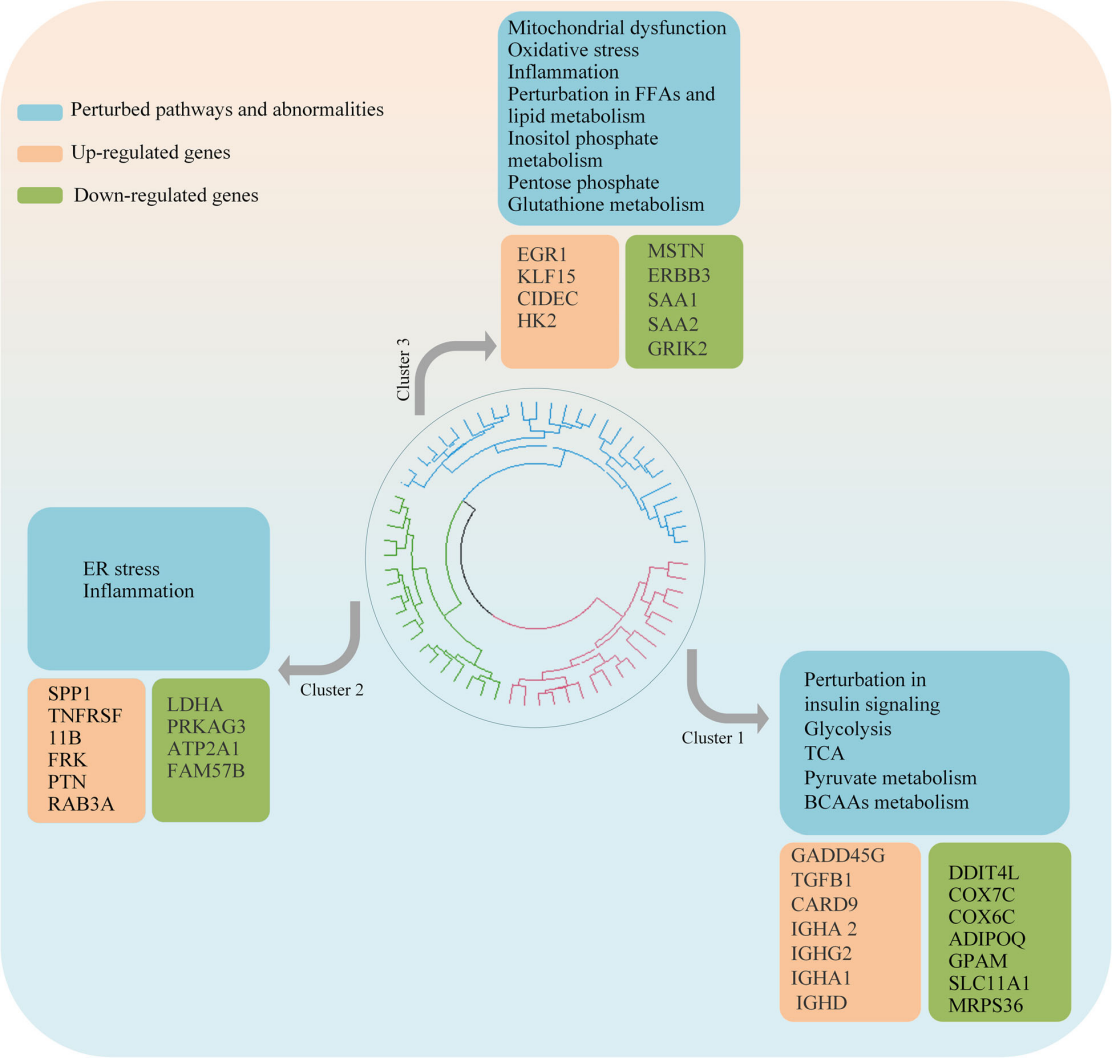
Hierarchical clustering of diabetic samples. The top three clusters were selected, and DEGs and perturbed pathways in each cluster compared to normal samples were found. Some of the specific dysregulated genes and pathways in each cluster are shown in the boxes. Green boxes show down-regulated genes, and peach boxes show up-regulated genes. The blue boxes show perturbed pathways and abnormalities in each cluster

In this section, the objective was to assess the possibility of the existence of different subtypes in the disease. We tried to answer the following questions: 1) Do the diabetic participants show different patterns of gene expression or not; and 2) Is it possible to categorize T2DM samples into distinct sub-groups with specific abnormalities in gene expression pattern? To answer the questions mentioned above, the unsupervised hierarchical clustering algorithm was exploited on diabetic samples using the measure of Euclidean distance and complete linkage method. The top three clusters were selected and studied (Fig. 3). Clusters 1 to 3 consist of 18, 18, and 27 individuals, respectively.

**Fig. 4:**
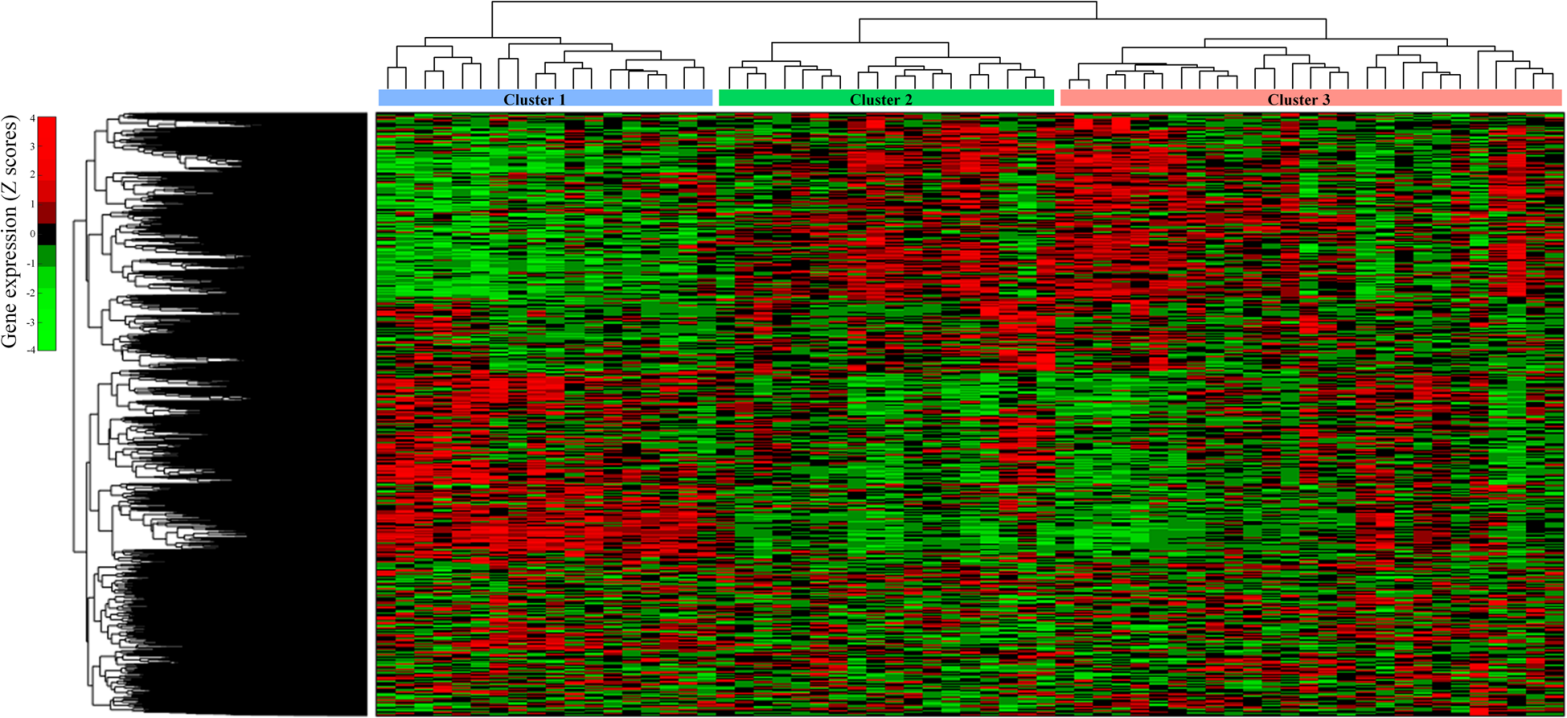
Heatmap representation of the gene expression pattern in three diabetic clusters. The columns of the heatmap represent diabetic individuals and the rows show standardized gene expression (Z scores). Higher expressions are shown in lighter red and lower expressions are shown in lighter green. Clusters with relevant dendrogram obtained from hierarchical clustering with Euclidean distance are shown at the top of columns in which blue, green, and red lines demonstrate clusters 1 to 3, respectively. Row dendrogram indicates the clustering of genes using complete linkage hierarchical clustering with Euclidean distance

To study biological differences between clusters, metabolic modeling of each cluster, and differential gene expression analysis were applied. It was found that differences in gene expression patterns and pathways between healthy and all newly diagnosed diabetic patients are low. Clustering of patients and analysis between each cluster and healthy individuals helped to find more DEGs and more perturbed pathways. Results showed that each cluster has specific dysregulated genes and pathways, which do not exist in the other two clusters. A heatmap representation of the gene expression in three clusters is shown in Fig. 4. In addition, pathway enrichment analysis of DEGs in each cluster was performed. The results can be found in Table S1–3 of Additional file 1.

The analysis demonstrated that among these three clusters, the first cluster has the most number of perturbed pathways and dysregulated genes. Dysregulation of several genes in cluster 1, including down-regulation of *DDIT4L*, subunits of cytochrome c oxidase, several mitochondrial genes, *ADIPOQ*, and up-regulation of several inflammatory genes such as *GADD45G*, *TGFB1*, *CARD9*, *IGHA2*, *IGHG2*, *IGHA1*, *IGHD*, and *MIF* genes were found. Down-regulation of several genes encoding mitochondrial genes and subunits of cytochrome c oxidase (COX) can reflect mitochondrial dysfunction and oxidative stress. Down-regulation of the adiponectin gene also was found in cluster 1. At the metabolic modeling level, perturbations in pathways related to inositol phosphate metabolism, pentose phosphate pathway, tyrosine metabolism, folate metabolism, acylglycerides metabolism, glutathione metabolism, ROS detoxification, glycerolipid metabolism, acyl-CoA hydrolysis, fatty acid activation, beta-oxidation of fatty acids, sphingolipid metabolism, glycerophospholipid metabolism, chondroitin/heparan sulfate metabolism, purine and pyrimidine metabolism, carnitine shuttle, TCA, oxidative phosphorylation, omega-3 and omega-6 fatty acid metabolism, and glycosphingolipid metabolism were observed.

Cluster 2 displayed no significant perturbed pathway, although, changes in the expression of various genes were observed. Overexpression of *SPP1*, *TNFRSF11B*, *FRK*, and down-regulation of *PRKAG3* and *ATP2A1* are some examples. We speculated that people in this group may be closer to the control group in respect of blood sugar levels. Thus, we compared the phenotypic features of people in each cluster with controls. Table 3 shows the average value of each feature in different clusters. In addition, the box plots of fasting glucose and fasting insulin values in each diabetic cluster and normoglycemic group are shown in Figures. S2 and S3 of Additional file 1. We also provided more information about differences of clinical features between each pair of clusters in Additional file 1 Table S4 and S5. This analysis revealed that this cluster is very close to the healthy state in terms of blood glucose and insulin levels.

**Table 3:** Subject characteristics for data. Fasting plasma glucose, fasting serum insulin, BMI, and waist/hip ratio (WHR) in each diabetic cluster and healthy group

	Healthy	Cluster 1	Cluster 2	Cluster 3	***P*** value
**Glucose (mmol/L)**	5.62 ± 0.3	7.17 ± 0.5	6.86 ± 0.5	7.39 ± 0.75	6.14e-43
**Insulin (mu/l)**	6.87 ± 3.3	10.19 ± 5.3	7.79 ± 3.9	12.93 ± 8.7	1.08e-06
**BMI**	26.35 ± 3.5	29.03 ± 5.0	28.58 ± 4.5	30.13 ± 5.5	1.96e-04
**WHR**	0.92 ± 0.08	0.99 ± 0.07	0.95 ± 0.06	1.02 ± 0.06	3.64e-08

All values are shown as means ± standard deviation

*P* values were calculated using ANOVA F-test

From these three clusters, cluster 3 has the least number of DEGs, although perturbations in the expression of various important genes like *MSTN*, *ERBB3*, *EGR1*, *CIDEC*, and *HK2* were found in this cluster. At the metabolic level, the perturbation in glucose metabolism was observed. Dysregulation of branched-chain amino acids (BCAAs) metabolism, glycolysis, pyruvate metabolism, tricarboxylic acid cycle, and glyoxylate/dicarboxylate metabolism and several exchange and transport reactions were found. The complete list of DEGs and perturbed reactions in each cluster can be found in Additional file 2.

## Discussion

### Supervised classification discriminates diabetic patients from healthy ones

In this study, gene expression data from newly diagnosed type 2 diabetic patients were analyzed using supervised and unsupervised machine learning approaches. At the supervised level, we aimed to identify a set of genes whose expressions were dysregulated in most patients and could potentially discriminate normoglycemic from T2DM individuals.

The gene set comprised of genes such as *FGFBP3*, *CERK*, *ETV5*, *E2F8*, *MAFB*, and non-coding RNAs, which may be used to study and develop novel T2DM treatments in the future. Noticeably, the injection of *FGFBP3* has been patented as a treatment for diabetes, obesity, and nonalcoholic fatty liver disease [23, 24]. It has been demonstrated that the single injection of *FGFBP3* regulates blood glucose level and keeps it at the normal range for more than 24 h. *CERK* plays an important role in inflammation-associated diseases [25]. It has been observed that *CERK* deficiency in *CERK*-null mice suppresses the elevation of obesity-mediated inflammatory cytokines and improves glucose intolerance [26]. Studies also have indicated the relationship between diet and obesity and *ETV5* gene expression, which participates in food intake control mechanisms [27].

Moreover, it has been found that impaired glucose tolerance in obese individuals is associated with the up-regulation of *E2F8*, which possibly is implicated in the progression of obesity, glucose intolerance, and its complications [28]. *MAFB* also has been linked to the metabolism and development of obesity and diabetes. The *MAFB*-deficient mice have exhibited higher body weights and a faster rate of increase in body weight than control mice [29]. Up-regulation of *MAFB* expression in human adipocytes has been correlated with adverse metabolic features and inflammation, which may lead to the development of insulin resistance [30]. In addition to the protein-encoding genes, we found that about 40% of top-ranked genes comprise non-coding RNAs, including pseudogenes and long non-coding RNAs. Recent studies have revealed that the deregulation of pseudogenes and lncRNAs can relate to diabetes [31, 32]. In the present analysis, more non-coding candidates were found that support the role of lncRNAs in complex diseases like diabetes. These non-coding RNAs can be functionally analyzed to understand their biological roles in the pathology of T2DM.

### Unsupervised classification of diabetic patients reveals the potential existence of molecular subtypes

The objective of analysis at the unsupervised level was to identify different gene expression patterns among T2DM patients, potentially leading to insulin resistance through different mechanisms. In this part, the diabetic samples were categorized into three clusters, and specific dysregulated genes and pathways in each cluster were found. This analysis shows that because of the heterogeneous and multifactorial nature of this disease, the gene expression dysregulations of all diabetic people are not necessarily the same. Thus, people can be clustered into different subgroups with different dysregulations in gene expression patterns. We attempted to model the subsequent effects of these gene expression dysregulations on their metabolisms. Although, we did not claim these transcriptional differences lead to the manifestation of different clinical features such as fasting glucose and insulin levels in these clusters. Moreover, we only investigated the potential existence of molecular subtypes in T2DM, and we did not introduce specific subtypes. Accurate subtyping requires more data from additional individuals and validation with an independent data set and experimental verification.

#### Cluster 1: mitochondrial dysfunction, oxidative stress, and inflammation

In cluster 1, perturbed pathways and dysregulated genes possibly represent perturbation of lipid and free fatty acids (FFAs) metabolism, inflammation, oxidative stress, and mitochondrial dysfunction. Perturbed pentose phosphate, folate metabolism, and glutathione metabolism as well as dysregulated genes such as *IGHA1* and *IGHA2*, *GADD45G*, and *DDIT4* exhibit inflammation and oxidative stress. The up-regulation of *IGHA1* and *IGHA2* may trigger an inflammatory cascade involving a neutrophilic response, phagocytosis, the oxidative burst, and subsequent tissue damage. Also, *GADD45G* plays the role of a stress sensor [33] which is overexpressed in this group. DNA damage and energy stress can also activate *DDIT4* expression; thus, this gene contributes to regulating reactive oxygen species [34]. Oxidative stress may impair mitochondrial function, which possibly leads to impairment of insulin sensitivity. Some evidence has supported the role of oxidative stress and mitochondrial dysfunction in the pathogenesis of insulin resistance and type 2 diabetes [35]. In diabetes mellitus, mitochondria are the major source of oxidative stress [35]. Free radicals can damage lipids, proteins, and DNA and play a role in diabetes complications. Down-regulated mitochondrial genes and perturbation in oxidative phosphorylation may demonstrate mitochondrial dysfunction in this cluster. Furthermore, MIF, which is a proinflammatory cytokine, is up-regulated in this cluster. A positive association has been reported between MIF plasma levels, FFAs concentration, and insulin resistance [36]. The perturbation of FFAs metabolism that possibly leads to an increase in FFAs was observed in this cluster. Evidence has demonstrated that FFAs can induce insulin resistance in skeletal muscle. FFAs may induce insulin resistance via mitochondrial dysfunction, increased ROS production and oxidative stress, and activation of inflammatory signals, which was observed in this cluster [37]. An increase in FFAs is associated with a decrease in adiponectin. ADIPOQ is mainly known as the adipokine, but the importance of adiponectin production in muscle cells has also been demonstrated [38]. This study also has reported an increased expression of adiponectin in response to rosiglitazone treatment in muscle cells and has confirmed the functional role of muscle adiponectin in insulin sensitivity. Adiponectin contributes to the glucose metabolism of muscle cells via increased insulin-induced serine phosphorylation of protein kinase B and inhibition of the inflammatory response [39]. Moreover, in this cluster, abnormalities in inositol phosphate metabolism with Myo-inositol deficiency was observed. Myo-inositol, one of the inositol isomers, participates in signal transduction and vesicle trafficking and associates with glucose utilization. Clinical reports have suggested that the administration of inositol supplements is a therapeutic approach in insulin resistance and improves glucose metabolism [40]. Figure S4 in Additional file 1 shows the overview of abnormalities in this cluster.

#### Cluster 2: ER-stress and inflammation

Surprisingly, no significant dysregulated pathway found in the second cluster. Therefore, we compared the phenotypic features of people in each cluster with healthy individuals. It was interesting that this cluster is very similar to the healthy state in respect of blood glucose and insulin levels. Therefore, people at this group may be at the early stage of diabetes onset, and there is still no apparent change in their metabolism. However, using differential gene expression analysis, the changes in the expression of non-metabolic genes (e.g. overexpression of *OPN*, *OPG*, *CHAC1*, *ERN1*, and down-regulation of *SERCA1)* were observed in this cluster. These genes are related to diabetes by promoting ER-stress and inflammation. OPN and OPG play roles in inflammation, insulin resistance, prediabetes, and diabetes. A recent study has demonstrated that OPN and OPG levels in pre-diabetic subjects are increased, and alterations in OPN and OPG might be involved in the pathogenesis of prediabetes and T2DM [41, 42]. Obese mice lacking osteopontin have shown improved whole-body glucose tolerance and insulin resistance, also with decreased markers of inflammation [43]. In addition, ER-stress can induce the expression of *OPN* and *OPG*. Recent pieces of evidence have supported the presence and role of ER stress in muscle [44–46]. In this cluster, SERCA1, which is an intracellular membrane-bound Ca^2+^-transport ATPase enzyme encoded by the *ATP2A1* gene was down-regulated. The dysregulation of SERCA promotes ER Stress [41]. SERCA1 resides in the sarcoplasmic or endoplasmic reticula of muscle cells and contributes to the modulation of cellular Ca^2+^ homeostasis within the physiological range. Lower SERCA expression may lead to reduced Ca^2+^ accumulation in the ER lumen and ER dysfunction. High luminal calcium concentration is essential for proper protein folding and processing. Ca^2+^ depletion can result in the accumulation of unfolded proteins and can trigger the unfolded protein response (UPR) and cell death [47]. High-fat diet and obesity induce ER stress in muscles and subsequently suppress insulin signaling [48]. Antidiabetic compounds such as azoramide and rosiglitazone, have been demonstrated to induce SERCA expression and increased accumulation of Ca^2+^ in ER [49, 50]. Schematic representation of abnormalities in cluster 2 is shown in Figure S5 of Additional file 1.

#### Cluster 3: perturbation in IRS-mediated insulin signaling

In cluster 3, the differential gene expression analysis revealed the perturbation in insulin signaling and inflammation. Results showed down-regulation of insulin-responsive genes, *HK2*, *EGR1*, and *CIDEC*, which verify insulin resistance through deficiency of insulin signaling. Furthermore, overexpression of *MSTN* and *ERBB3* was found. Myostatin has been shown to induce insulin resistance by degrading IRS1 proteins [51] and diminishing insulin-induced IRS1 tyrosine phosphorylation, thus interrupting insulin signaling cascade [52]. In addition, treating HeLa cells with myostatin has suppressed *HK2* expression [53]. Evidence has revealed that stress-induced transactivation of ERBB2/ERBB3 receptors triggers a PI3K cascade leading to the serine phosphorylation of IRS proteins [54, 55]. Overexpression of *ERBB3* may enhance PI3K activity and implicating ERBB proteins in stress-induced insulin resistance. Taken together, *MSTN* and *ERBB3* can lead to serine phosphorylation of IRS, reducing tyrosine phosphorylation of IRS and degradation of them. Since expressions of insulin-regulated genes are positively correlated with insulin sensitivity, down-regulation of *HK2*, *EGR1*, and *CIDEC* genes in this group possibly verify insulin resistance through deficiency of insulin signaling. In addition, at the metabolic analysis, lower phosphorylation of glucose with the subsequent perturbation in glycolysis and TCA pathways was observed. Moreover, dysmetabolism of branched-chain amino acids was observed at metabolic analysis. A mechanism involved leucine-mediated activation of the mammalian target of rapamycin complex 1 (mTORC1) has been proposed to link higher levels of BCAAs and T2DM [56]. This activation results in the serine phosphorylation of IRS1 and IRS2 and subsequent uncoupling of insulin signaling at an early stage. A brief representation of abnormalities in this cluster is shown in Figure S6 of Additional file 1.

### The cluster-based study can improve understanding of T2DM

Our analysis showed that at the early stage of diabetes, associated changes at the gene expression level in skeletal muscle are low, compared to healthy subjects. Moreover, the clustering of patients leads to the identification of the abnormalities that are usually hidden in cohort studies. For example, dysregulation of genes such as *MIF*, *ATP2A1*, *GADD45G*, *EEF2*, *EGR1*, *CIDEC*, and *MSTN*, and perturbations in several reactions implicated in BCAAs metabolism, folate metabolism, and pentose phosphate were only observed in our cluster-based analysis. In a cohort study, a sample consists of several subjects is gathered and is examined (Figure S7 of Additional file 1). This makes it possible to see only an approximate average of the features in the samples and as a result, some of the abnormalities are covered in this way. In a cluster-based study, a collected sample in a cohort study is broken down into the sub-groups so that the members within each subgroup have the most similarity and differ from the members of the outer sub-groups. Each sub-group will be analyzed individually (e.g., here we divided the diabetic group into three sub-groups). The cluster-based analysis in this study led to find more dysregulated genes and pathways that are specific in each cluster. Therefore, for a progressive and heterogenic disease like T2DM, applying a cluster-based study will enhance our understanding of the factors involved in the disease pathogenesis. Focusing on homogeneous sub-groups in a heterogenic disease such as T2DM may improve the success of therapeutic strategies.

## Conclusion

In this study, the changes in gene expression patterns of newly diagnosed diabetic patients were analyzed using supervised and unsupervised classification methods. Using only gene expression data, it is possible to discriminate T2DM individuals from healthy controls with approximately 90% accuracy. Clustering of diabetic patients according to their gene expression patterns and subsequent more in-depth analysis of each cluster unraveled specific abnormalities leading to insulin resistance in each cluster. Based on the observed results in this work, it seems that the disease has the potential to be subtyped based on the gene expression patterns. This is a pilot study, and further empirical analysis is still needed to confirm our findings. We propose that using the unsupervised clustering of diabetic patients in combination with their real clinical profiles helps to find significant molecular subtypes of T2DM with specific abnormalities. This approach potentially will lead to better therapeutic measures in each subtype in the future.

## Supplementary information

**Additional file 1: Figures. S1–7.** show bar plot of SVM classification evaluation, boxplots related to individuals characteristics in each cluster and schematic representation of abnormalities in each cluster. **Tables S1–3.** related to KEGG pathway enrichment analysis of each cluster.

**Additional file 2.** The complete list of differentially expressed genes in each cluster, top-ranked genes with Ensemble ID, perturbed reactions obtained from metabolic modeling in each cluster.

## Data Availability

The dataset analyzed during the current study are available through the repository’s data access request procedure in the database of Genotypes and Phenotypes (dbGaP) with the accession code phs001068.v1.p1, https://www.ncbi.nlm.nih.gov/projects/gap/cgi-bin/study.cgi?study_id=phs001068.v1.p1. The codes used in this article are available at https://github.com/Maryamkhn/T2DM_potential_subtyping.

## Abbreviations

T2DM: Type 2 diabetes mellitus
SVM: Support vector machine
GA: Genetic algorithm
KNN: K-nearest neighbor
NN: Neural network
NB: Naïve Bayes
RF: Random forest
AUC: Area under the ROC curve
ACC: Accuracy
GEMs: Genome-scale metabolic models
FVA: Flux variability analysis
DEGs: Differentially expressed genes
FFAs: Free fatty acids
UPR: Unfolded protein response

## References

[1] DeFronzo RA, Ferrannini E, Groop L, Henry RR, Herman WH, Holst JJ, Hu FB, Kahn CR, Raz I, Shulman GI (2015). Type 2 diabetes mellitus. Nat Re Dis Primers.

[2] Björnholm M, Zierath J. Insulin signal transduction in human skeletal muscle: identifying the defects in Type II diabetes. Biochem Soc Trans. 2005;33(2):354–7. 10.1042/BST0330354 15787605

[3] DeFronzo RA (2009). From the triumvirate to the ominous octet: a new paradigm for the treatment of type 2 diabetes mellitus. Diabetes.

[4] Yaribeygi H, Farrokhi FR, Butler AE, Sahebkar A (2019). Insulin resistance: review of the underlying molecular mechanisms. J Cell Physiol.

[5] Nogiec C, Burkart A, Dreyfuss JM, Lerin C, Kasif S, Patti M-E (2015). Metabolic modeling of muscle metabolism identifies key reactions linked to insulin resistance phenotypes. Mol Metab.

[6] Bordbar A, Feist AM, Usaite-Black R, Woodcock J, Palsson BO, Famili I (2011). A multi-tissue type genome-scale metabolic network for analysis of whole-body systems physiology. BMC Syst Biol.

[7] Väremo L, Scheele C, Broholm C, Mardinoglu A, Kampf C, Asplund A, Nookaew I, Uhlén M, Pedersen BK, Nielsen J (2015). Proteome-and transcriptome-driven reconstruction of the human myocyte metabolic network and its use for identification of markers for diabetes. Cell Rep.

[8] Väremo L, Nookaew I, Nielsen J (2013). Novel insights into obesity and diabetes through genome-scale metabolic modeling. Front Physiol.

[9] Scott LJ, Erdos MR, Huyghe JR, Welch RP, Beck AT, Wolford BN, Chines PS, Didion JP, Narisu N, Stringham HM. The genetic regulatory signature of type 2 diabetes in human skeletal muscle. Nat Commun. 2016;7(1):1–12. 10.1038/ncomms11764 PMC4931250 27353450

[10] Ching T, Huang S, Garmire LX (2014). Power analysis and sample size estimation for RNA-Seq differential expression. Rna.

[11] Soneson C, Delorenzi M (2013). A comparison of methods for differential expression analysis of RNA-seq data. BMC Bioinformatics.

[12] Love MI, Huber W, Anders S (2014). Moderated estimation of fold change and dispersion for RNA-seq data with DESeq2. Genome Biol.

[13] Dillies M-A, Rau A, Aubert J, Hennequet-Antier C, Jeanmougin M, Servant N, Keime C, Marot G, Castel D, Estelle J (2013). A comprehensive evaluation of normalization methods for Illumina high-throughput RNA sequencing data analysis. Brief Bioinform.

[14] Kuleshov MV, Jones MR, Rouillard AD, Fernandez NF, Duan Q, Wang Z, Koplev S, Jenkins SL, Jagodnik KM, Lachmann A (2016). Enrichr: a comprehensive gene set enrichment analysis web server 2016 update. Nucleic Acids Res.

[15] Demšar J, Curk T, Erjavec A, Gorup Č, Hočevar T, Milutinovič M, Možina M, Polajnar M, Toplak M, Starič A (2013). Orange: data mining toolbox in python. J Mach Learn Res.

[16] Orth JD, Thiele I, Palsson BØ (2010). What is flux balance analysis?. Nat Biotechnol.

[17] Mardinoglu A, Agren R, Kampf C, Asplund A, Nookaew I, Jacobson P, Walley AJ, Froguel P, Carlsson LM, Uhlen M. Integration of clinical data with a genome‐scale metabolic model of the human adipocyte. Mol Syst Biol. 2013;9(1):649. 10.1038/msb.2013.5 PMC3619940 23511207

[18] Mardinoglu A, Agren R, Kampf C, Asplund A, Uhlen M, Nielsen J (2014). Genome-scale metabolic modelling of hepatocytes reveals serine deficiency in patients with non-alcoholic fatty liver disease. Nat Commun.

[19] Colijn C, Brandes A, Zucker J, Lun DS, Weiner B, Farhat MR, Cheng T-Y, Moody DB, Murray M, Galagan JE. Interpreting expression data with metabolic flux models: predicting Mycobacterium tuberculosis mycolic acid production. PLoS Comput Biol. 2009;5(8):e1000489. 10.1371/journal.pcbi.1000489 PMC2726785 19714220

[20] Hadi M, Marashi S-A (2014). Reconstruction of a generic metabolic network model of cancer cells. Mol BioSyst.

[21] Chénard T, Guénard F, Vohl M-C, Carpentier A, Tchernof A, Najmanovich RJ (2017). Remodeling adipose tissue through in silico modulation of fat storage for the prevention of type 2 diabetes. BMC Syst Biol.

[22] Heirendt L, Arreckx S, Pfau T, Mendoza SN, Richelle A, Heinken A, Haraldsdóttir HS, Wachowiak J, Keating SM, Vlasov V. Creation and analysis of biochemical constraint-based models using the COBRA Toolbox v. 3.0. Nat Protoc. 2019;14(3):639–702. 10.1038/s41596-018-0098-2 PMC6635304 30787451

[23] Garman KA. Fibroblast growth factor binding protein 3: a novel target for glucose intolerance and nonalcoholic fatty liver disease treatment. Washington, D.C.: Georgetown University; 2018.

[24] Wellstein A (2018). Compositions and Treatments of Metabolic Disorders Using FGF Binding Protein 3. US Patent App.

[25] Yu WL, Sun Y (2015). CERK inhibition might be a good potential therapeutic target for diseases. Br J Pharmacol.

[26] Mitsutake S, Date T, Yokota H, Sugiura M, Kohama T, Igarashi Y (2012). Ceramide kinase deficiency improves diet-induced obesity and insulin resistance. FEBS Lett.

[27] Boender AJ, Van Rozen AJ, Adan RA (2012). Nutritional state affects the expression of the obesity-associated genes Etv5, Faim2, Fto, and Negr1. Obesity.

[28] Minchenko O, Bashta Y, Minchenko D, Ratushna O (2016). Glucose tolerance in obese men is associated with dysregulation of some angiogenesis-related gene expressions in subcutaneous adipose tissue. Fiziol Zh.

[29] Tran MTN, Hamada M, Nakamura M, Jeon H, Kamei R, Tsunakawa Y, Kulathunga K, Lin YY, Fujisawa K, Kudo T (2016). MafB deficiency accelerates the development of obesity in mice. FEBS Open Bio.

[30] Pettersson AM, Acosta JR, Björk C, Krätzel J, Stenson B, Blomqvist L, Viguerie N, Langin D, Arner P, Laurencikiene J (2015). MAFB as a novel regulator of human adipose tissue inflammation. Diabetologia.

[31] Zhang N, Geng T, Wang Z, Zhang R, Cao T, Camporez JP, Cai S-Y, Liu Y, Dandolo L, Shulman GI. Elevated hepatic expression of H19 long noncoding RNA contributes to diabetic hyperglycemia. JCI Insight. 2018;3(10):e120304. 10.1172/jci.insight.120304 PMC6012507 29769440

[32] Chiefari E, Iiritano S, Paonessa F, Le Pera I, Arcidiacono B, Filocamo M, Foti D, Liebhaber SA, Brunetti A (2010). Pseudogene-mediated posttranscriptional silencing of HMGA1 can result in insulin resistance and type 2 diabetes. Nat Commun.

[33] Liebermann DA, Hoffman B (2007). Gadd45 in the response of hematopoietic cells to genotoxic stress. Blood Cell Mol Dis.

[34] Ellisen LW, Ramsayer KD, Johannessen CM, Yang A, Beppu H, Minda K, Oliner JD, McKeon F, Haber DA (2002). REDD1, a developmentally regulated transcriptional target of p63 and p53, links p63 to regulation of reactive oxygen species. Mol Cell.

[35] Asmat U, Abad K, Ismail K (2016). Diabetes mellitus and oxidative stress—a concise review. Saudi Pharm J.

[36] Tripathy D, Mohanty P, Dhindsa S, Syed T, Ghanim H, Aljada A, Dandona P (2003). Elevation of free fatty acids induces inflammation and impairs vascular reactivity in healthy subjects. Diabetes.

[37] Rachek LI. Free fatty acids and skeletal muscle insulin resistance. Prog Mol Biol Transl. vol. 121: Elsevier; 2014. p. 267–92. 10.1016/B978-0-12-800101-1.00008-9 24373240

[38] Liu Y, Chewchuk S, Lavigne C, Brûlé S, Pilon G, Houde V, Xu A, Marette A, Sweeney G (2009). Functional significance of skeletal muscle adiponectin production, changes in animal models of obesity and diabetes, and regulation by rosiglitazone treatment. Am J Physiol Endocrinol Metab.

[39] Wang CH, Wang CC, Huang HC, Wei YH (2013). Mitochondrial dysfunction leads to impairment of insulin sensitivity and adiponectin secretion in adipocytes. FEBS J.

[40] Bevilacqua A, Bizzarri M. Inositols in insulin signaling and glucose metabolism. Int J Endocrinol. 2018;2018. 10.1155/2018/1968450 PMC6286734 30595691

[41] Daniele G, Winnier D, Mari A, Bruder J, Fourcaudot M, Pengou Z, Hansis-Diarte A, Jenkinson C, Tripathy D, Folli F (2018). The potential role of the osteopontin–osteocalcin–osteoprotegerin triad in the pathogenesis of prediabetes in humans. Acta Diabetol.

[42] Kahles F, Findeisen HM, Bruemmer D (2014). Osteopontin: a novel regulator at the cross roads of inflammation, obesity and diabetes. Mol Metab.

[43] Chapman J, Miles PD, Ofrecio JM, Neels JG, Joseph GY, Resnik JL, Wilkes J, Talukdar S, Thapar D, Johnson K (2010). Osteopontin is required for the early onset of high fat diet-induced insulin resistance in mice. PLoS One.

[44] Deldicque L, Cani PD, Philp A, Raymackers J-M, Meakin PJ, Ashford ML, Delzenne NM, Francaux M, Baar K (2010). The unfolded protein response is activated in skeletal muscle by high-fat feeding: potential role in the downregulation of protein synthesis. Am J Physiol Endocrinol Metab.

[45] Deldicque L, Hespel P, Francaux M (2012). Endoplasmic reticulum stress in skeletal muscle: origin and metabolic consequences. Exerc Sport Sci Rev.

[46] Rayavarapu S, Coley W, Van der Meulen JH, Cakir E, Tappeta K, Kinder TB, Dillingham BC, Brown KJ, Hathout Y, Nagaraju K (2013). Activation of the ubiquitin proteasome pathway in a mouse model of inflammatory myopathy: a potential therapeutic target. Arthritis Rheum.

[47] Arruda AP, Hotamisligil GS (2015). Calcium homeostasis and organelle function in the pathogenesis of obesity and diabetes. Cell Metab.

[48] Koh H-J, Toyoda T, Didesch MM, Lee M-Y, Sleeman MW, Kulkarni RN, Musi N, Hirshman MF, Goodyear LJ (2013). Tribbles 3 mediates endoplasmic reticulum stress-induced insulin resistance in skeletal muscle. Nat Commun.

[49] Shah R, Gonzales F, Golez E, Augustin D, Caudillo S, Abbott A, Morello J, McDonough P, Paolini P, Shubeita H (2005). The antidiabetic agent rosiglitazone upregulates SERCA2 and enhances TNF-α-and LPS-induced NF-κB-dependent transcription and TNF-α-induced IL-6 secretion in ventricular myocytes. Cell Physiol Biochem.

[50] Fu S, Yalcin A, Lee GY, Li P, Fan J, Arruda AP, Pers BM, Yilmaz M, Eguchi K, Hotamisligil GS (2015). Phenotypic assays identify azoramide as a small-molecule modulator of the unfolded protein response with antidiabetic activity. Sci Transl Med.

[51] Bonala S, Lokireddy S, McFarlane C, Patnam S, Sharma M, Kambadur R (2014). Myostatin induces insulin resistance via casitas B-lineage lymphoma b (Cblb)-mediated degradation of insulin receptor substrate 1 (IRS1) protein in response to high calorie diet intake. J Biol Chem.

[52] Liu XH, Bauman WA, Cardozo CP (2018). Myostatin inhibits glucose uptake via suppression of insulin-dependent and-independent signaling pathways in myoblasts. Physiol Rep.

[53] Liu Y, Cheng H, Zhou Y, Zhu Y, Bian R, Chen Y, Li C, Ma Q, Zheng Q, Zhang Y (2013). Myostatin induces mitochondrial metabolic alteration and typical apoptosis in cancer cells. Cell Death Dis.

[54] Hemi R, Paz K, Wertheim N, Karasik A, Zick Y, Kanety H. Transactivation of ErbB2 and ErbB3 by tumor necrosis factor-α and anisomycin leads to impaired insulin signaling through serine/threonine phosphorylation of IRS proteins. J Biol Chem. 2002;277(11):8961–9. 10.1074/jbc.M109391200 11779863

[55] Hemi R, Yochananov Y, Barhod E, Kasher-Meron M, Karasik A, Tirosh A, Kanety H. p38 mitogen-activated protein kinase-dependent transactivation of ErbB receptor family: a novel common mechanism for stress-induced IRS-1 serine phosphorylation and insulin resistance. Diabetes. 2011;60(4):1134–45. 10.2337/db09-1323 PMC3064087 21386087

[56] Lynch CJ, Adams SH (2014). Branched-chain amino acids in metabolic signalling and insulin resistance. Nat Rev Endocrinol.

