# Supplementary material for: Unraveling the molecular heterogeneity in type 2 diabetes: a potential subtype discovery followed by metabolic modeling
Source: BMC Med Genomics. 2020 Aug 24;13:119. doi: 10.1186/s12920-020-00767-0 (PMC7444195; doi:10.1186/s12920-020-00767-0)
Supplement: Supplementary file 1 — Additional file 1: Figures. S1–7. show bar plot of SVM classification evaluation, boxplots related to individuals characteristics in each cluster and schematic representation of abnormalities in each cluster. Tables S1–3. related to KEGG pathway enrichment analysis of each cluster. [file 12920_2020_767_MOESM1_ESM.docx]

Unraveling the Molecular Heterogeneity in type 2 Diabetes: A Potential Subtype Discovery Study Followed by Metabolic Modeling

Maryam Khoshnejat^1, 4^, Kaveh Kavousi^1, 4, *^, Ali Mohammad Banaei- Moghaddam^2, 4^, Ali Akbar Moosavi-Movahedi^3, 4^

^1^ Laboratory of Complex Biological Systems and Bioinformatics (CBB), Department of Bioinformatics, Institute of Biochemistry and Biophysics (IBB), University of Tehran, Tehran, Iran

^2^ Laboratory of Genomics and Epigenomics (LGE), Department of Biochemistry, Institute of Biochemistry and Biophysics (IBB), University of Tehran, Tehran, Iran

^3^ Institute of Biochemistry and Biophysics, University of Tehran, Tehran, Iran

^4^ The UNESCO Chair on Interdisciplinary Research in Diabetes, Institute of Biochemistry and Biophysics (IBB), University of Tehran, Tehran, Iran


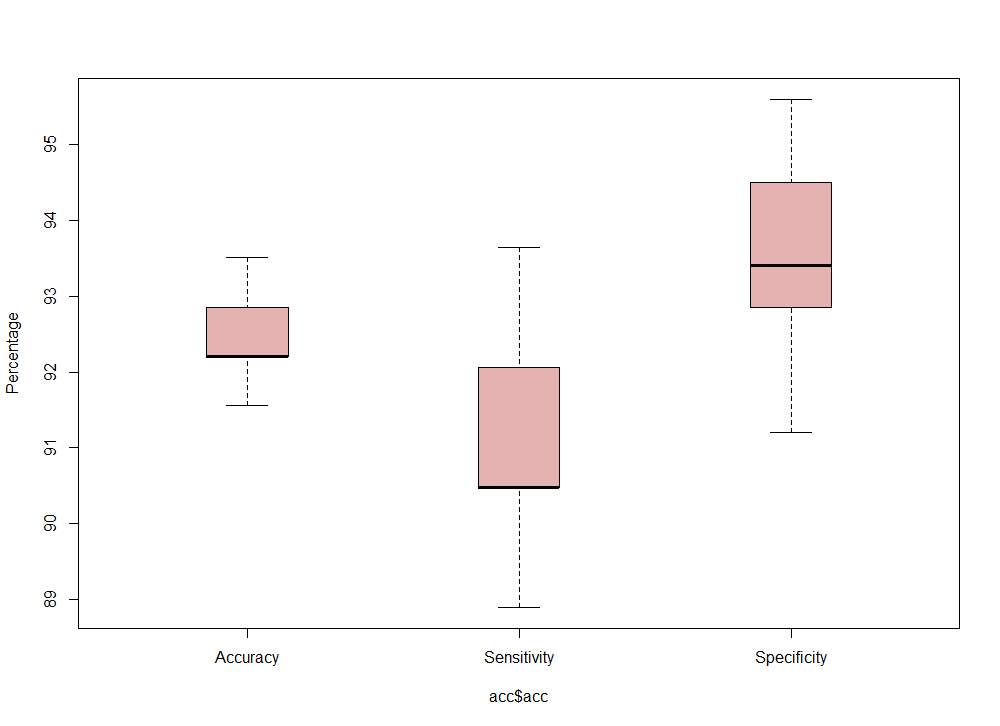


Figure S1. Accuracy, sensitivity and specificity of SVM classifier for 100 repetition of classification with 10-fold cross-validation


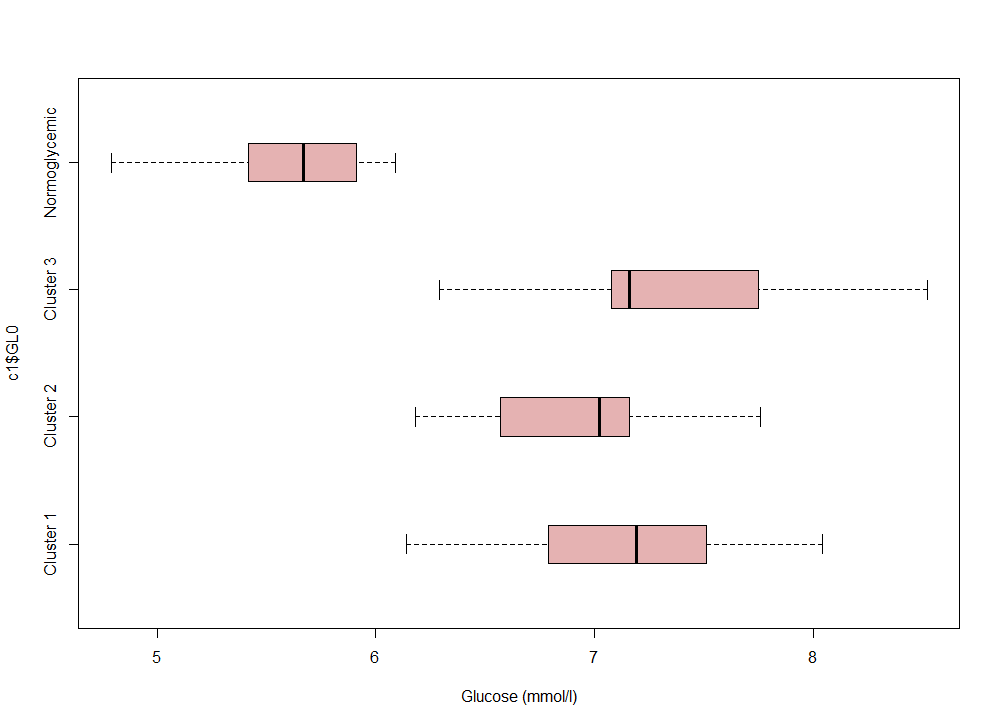


Figure S2. Box plot of fasting glucose values in each diabetic cluster and normoglycemic group.


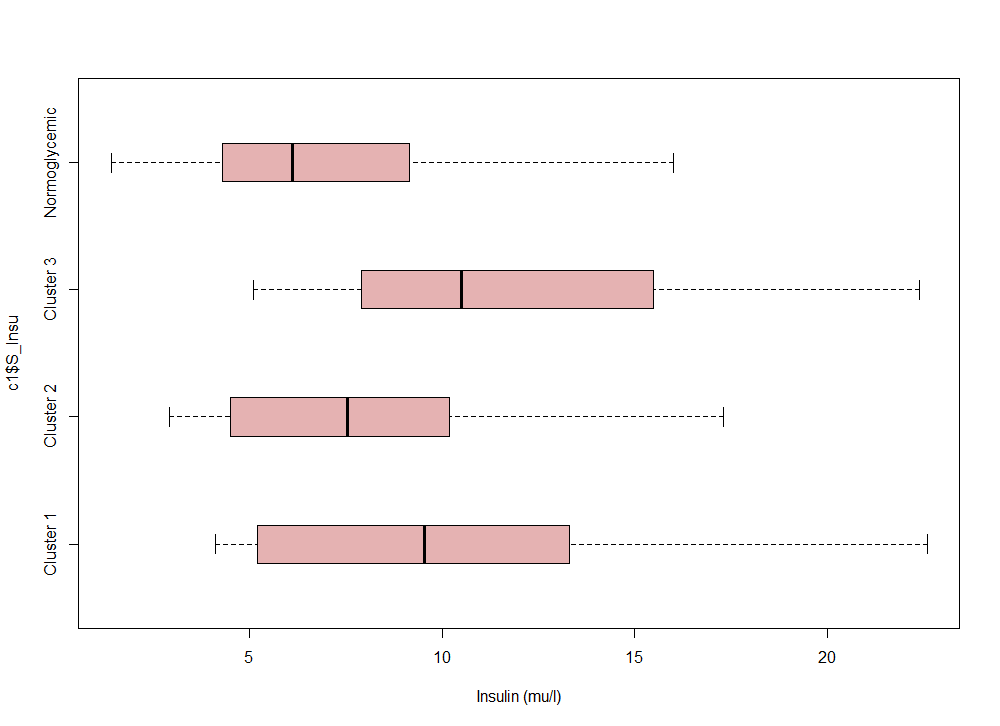


Figure S3. Box plot of fasting insulin values in each diabetic cluster and normoglycemic group.


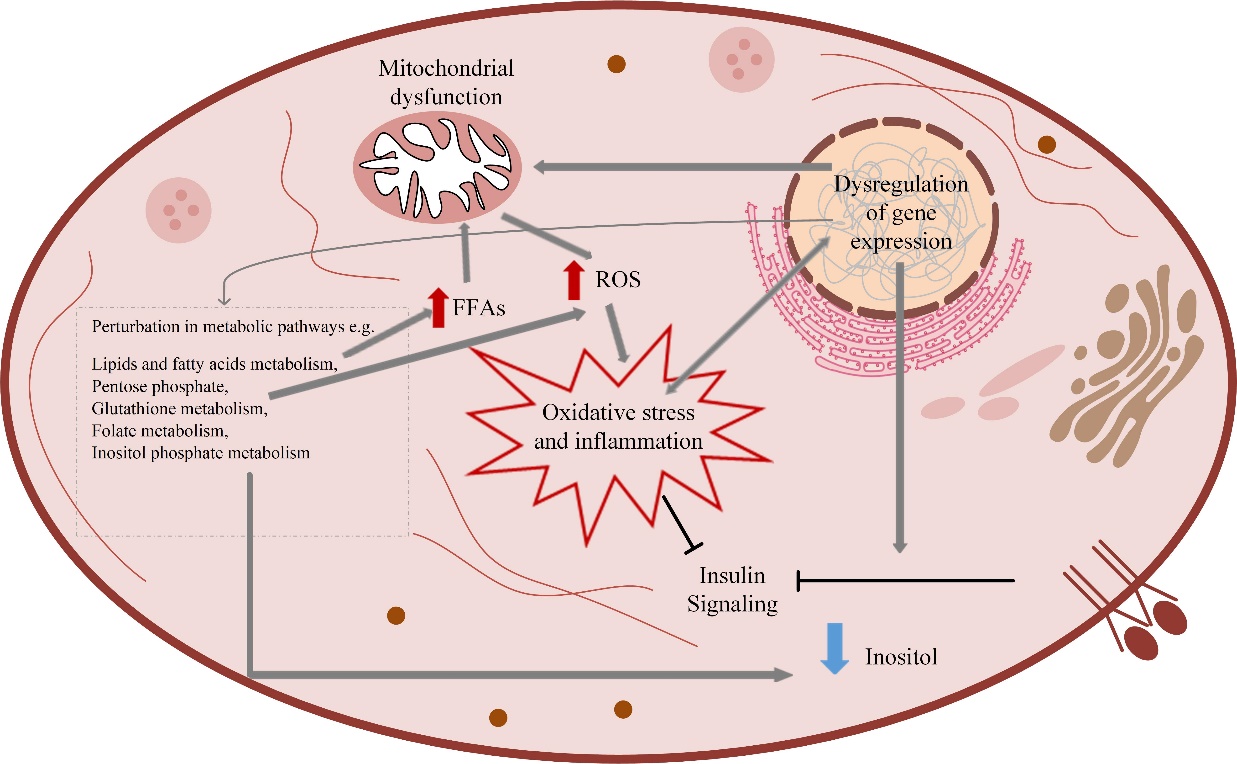

Figure S4. Schematic representation of abnormalities in cluster 1


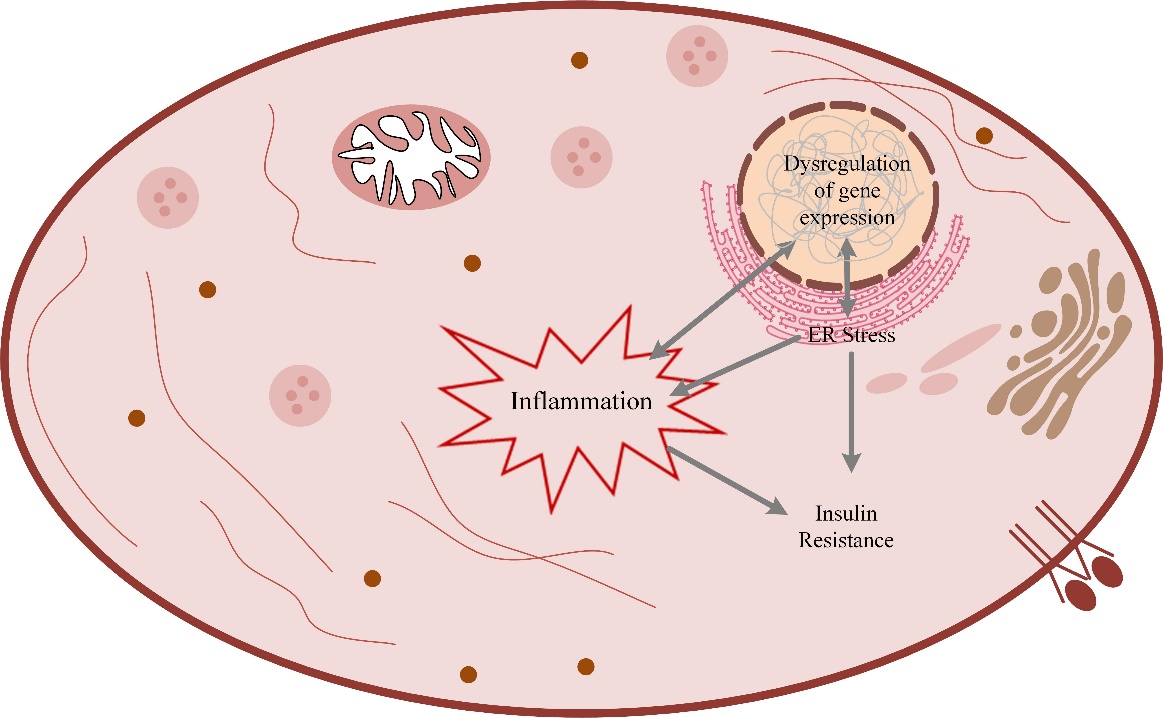


Figure S5. Schematic representation of abnormalities in cluster 2


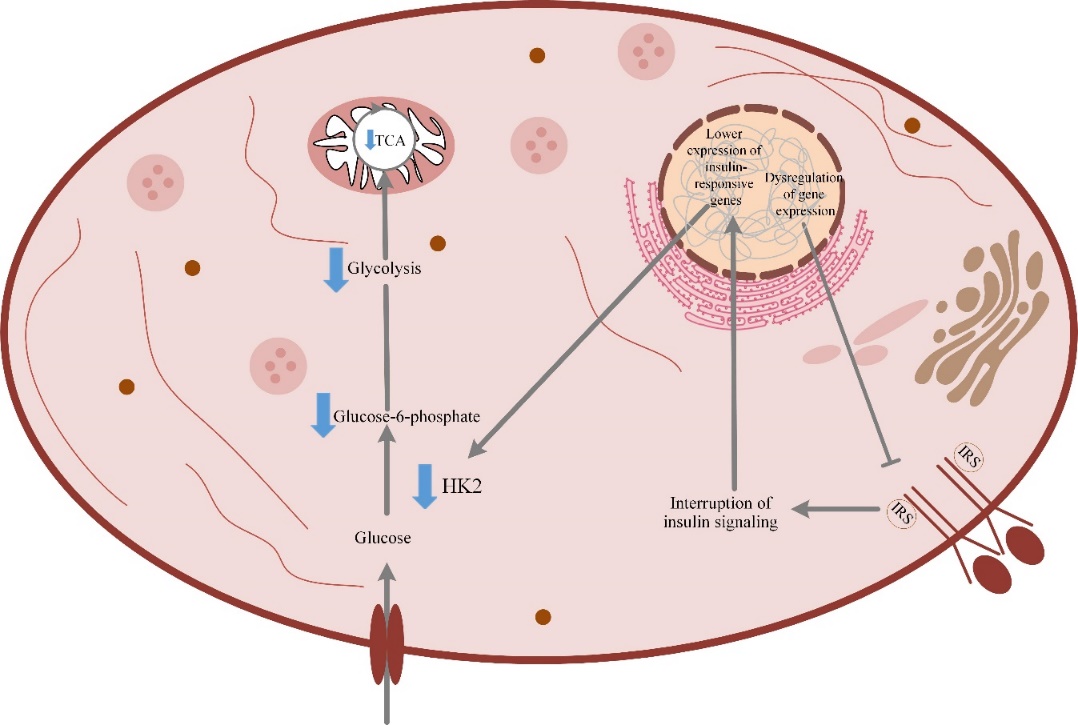


Figure S6. Schematic representation of abnormalities in cluster 3


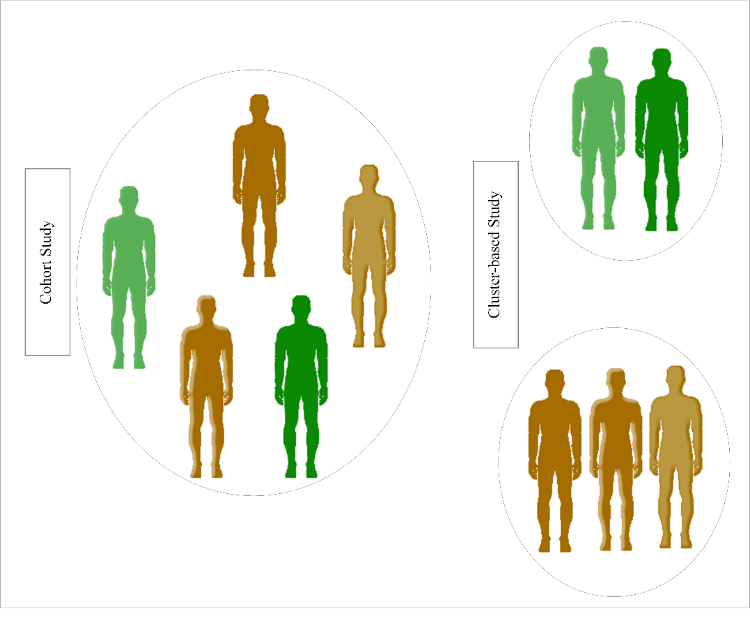

Figure S7. Cluster-based study versus cohort study. In a cohort study, a sample consists of several subjects is gathered and examined. In a cluster-based study, a sample that has been collected in a cohort study is broken down into the sub-groups so that the members within each subgroup have the most similarity and differ from the members of the outer sub-groups.

| **Name** | **p-value** |
| --- | --- |
| Starch and sucrose metabolism | 0.00015 |
| Phenylalanine metabolism | 0.024 |
| Mannose type O-glycan biosynthesis | 0.042 |
| Butanoate metabolism | 0.06 |
| Adipocytokine signaling pathway | 0.076 |
| Tyrosine metabolism | 0.094 |

Table S1. KEGG pathway enrichment of differentially expressed genes in cluster 1

| **Name** | **p-value** |
| --- | --- |
| ECM-receptor interaction | 0.015 |
| Phenylalanine metabolism | 0.037 |
| Mannose type O-glycan biosynthesis | 0.05 |
| Focal adhesion | 0.07 |
| Tyrosine metabolism | 0.07 |

Table S2. KEGG pathway enrichment of differentially expressed genes in cluster 2

| **Name** | **p-value** |
| --- | --- |
| Fructose and mannose metabolism | 0.009 |
| Calcium signaling pathway | 0.01 |
| Type II diabetes mellitus | 0.018 |
| MAPK signaling pathway | 0.04 |
| cAMP signaling pathway | 0.07 |
| HIF-1 signaling pathway | 0.07 |
| PI3K-Akt signaling pathway | 0.075 |

Table S3. KEGG pathway enrichment of differentially expressed genes in cluster 3

| ANOVA test between 3 clusters | P-value |
| --- | --- |
| Glucose | 0.03 |
| Insulin | 0.05 |
| BMI | 0.58 |
| Height | 0.02 |
| Waist | 0.07 |
| WHR | 0.003 |
| Age | 0.17 |
| Smoke | 0.7 |

Table S4. ANOVA test of clinical features between 3 clusters

|  | Cluster1_Cluster2 | Cluster1_Cluster3 | Cluster2_Cluster3 |
| --- | --- | --- | --- |
| Glucose | 0.0913 | 0.2581 | 0.009 |
| Insulin | 0.134 | 0.1966 | 0.0103 |
| BMI | 0.7788 | 0.4925 | 0.3084 |
| WHR | 0.0623 | 0.1516 | 0.0008 |
| Height | 0.0372 | 0.9636 | 0.0211 |
| Waist | 0.3616 | 0.1854 | 0.022 |
| Age | 0.107 | 0.3186 | 0.2683 |
| Smoke | 1 | 0.4957 | 0.4957 |

Table S5. Clinical features differences between each pair of clusters. P-value obtained from t-test between clusters
